# Elementary School-Aged Children’s and Parents’ Report of Health-Related Quality of Life and Relationships with Lifestyle Measures: A Cross-Sectional Study

**DOI:** 10.3390/nu15051264

**Published:** 2023-03-03

**Authors:** Soili Alanne, Ella Koivuniemi, Eliisa Löyttyniemi, Kirsi Laitinen

**Affiliations:** 1 Department of Clinical Nutrition, Wellbeing Services of County of South Ostrobothnia, 60220 Seinäjoki, Finland; 2 Institute of Biomedicine, Research Centre for Integrative Physiology and Pharmacology, University of Turku, 20014 Turku, Finland; 3 Functional Foods Forum (FFF), University of Turku, 20014 Turku, Finland; 4 Department of Biostatistics, University of Turku, 20014 Turku, Finland; 5 Department of Obstetrics and Gynecology, Turku University Hospital, 20521 Turku, Finland

**Keywords:** health-related-quality of life, children, Pediatric Quality of Life Inventory^TM^ 4.0, physical activity, diet quality

## Abstract

Supporting a child’s health-promoting lifestyle is an investment in their future health and health-related quality of life (HRQoL). Particularly children with overweight and obesity may be at an increased risk of a poor HRQoL. Currently, a comprehensive evaluation of lifestyle factors and age in relation to HRQoL in healthy children and, further, separate child and parental proxy-reports of HRQoL are lacking. The aims of this cross-sectional study in Finland are to compare healthy elementary school-aged children’s and parents’ reports of the child‘s HRQoL, and to view them in relation to lifestyle markers. The HRQoL was measured with Pediatric Quality of Life Inventory^TM^ 4.0, and the following lifestyle markers: leisure-time physical activity as MET, diet quality via a validated index (ES-CIDQ), sleeping time and screen time by questionnaires. Furthermore, age and BMI were recorded. Data were obtained from 270 primary school-aged children (6–13 years). Female gender, the child’s older age (8–13 years), high physical activity level and less screen time were strong predictors of a higher HRQoL in both the child’s and parental proxy-reports. Means to promote healthy lifestyles should be particularly targeted to young children, especially boys, and new ways to promote physical activity and other forms of free-time activities should be sought.

## 1. Introduction

Influencing in a positive way a child’s lifestyle is an investment in their future health. Therefore, health-promoting habits such as encouraging healthy eating, participating in physical activity, ensuring adequate hours of sleep, and a low amount of sedentary behaviours should be supported from early childhood onwards. According to the Finnish Current Care Guideline for obesity in children, adolescents, and adults, the promotion of healthy lifestyles is crucial for both the prevention and treatment of obesity [1]. In global terms, the prevalence of childhood obesity has increased and remains high in many countries [2]. In Finland, about one in four preschool-aged boys (24%) and every seventh girl (15%) were at least overweight, and by the time they reach elementary school, the prevalences increased to 28% in boys and 18% in girls [3].

Children are prone to be influenced by their everyday surroundings, not only by their family, friends, peers, but also by the media. Satisfaction with life reflects how a person in their social surroundings experiences the quality of their life against peers, and the children do not represent an exception. The health-related quality of life (HRQoL) is defined as the subjective assessment of the impact of disease and treatment across the physical, psychological, social, and somatic domains of functioning and wellbeing [4]. The HRQoL measures are generic questionnaires intended to obtain a subjective opinion by the respondents about their own health and wellbeing. It is important to take children‘s views into account when making decisions that affect them. The younger the children are, the more relevant it is to have access to a parallel parental proxy report of their child’s HRQoL [5]. Although studies are few, it has been noted that there are differences between children’s and parents’ reports; these vary according to the child’s age and gender [6]. The extent of agreement between the child’s and the parent’s reports has been shown to be affected by the child’s age, the domains investigated, and the parent’s own quality of life [7]. For example, parents tend to underestimate their child’s HRQoL when they have a congenital health condition [8] or a chronic illness [9]. Differences between boys’ and girls’ reports (aged 5 to 18 years) have also been detected; girls report their HRQoL as poorer than boys [10]. When comparing mean total scores of HRQoL across studies, children below the age of seven and adolescents above the age of 12 had higher HRQoL scores and thus better HRQoL [10].

Several previously published papers have focused predominantly on obese and overweight children [11,12]; they have revealed that obesity contributes to a lower HRQoL [11,13], lower physical, and psychosocial HRQoL [11]. However, the extent to which lifestyle factors, particularly diet intake, contribute to HRQoL were not inspected in these studies. Only recently, eating behaviours and especially adherence to the Mediterranean diet have been considered [14,15] There are also some studies which have focused on healthy children, regardless of their body weight. A high amount of screen time, a short sleep duration, and low physical activity were reported to be linked with poor HRQoL [16]. A systematic review evaluated the studies that had reported associations between physical activity, sedentary behaviours, and HRQoL in children and adolescents, and concluded that a higher level of physical activity with less time being spent on sedentary behaviours was associated with increased HRQoL among children and adolescents [17].

With respect to diet, a good diet quality and healthy dietary behaviours were associated with increased HRQoL in children [14] and adolescents [18]. There are, however, very few reports in which all important lifestyle factors and age have been evaluated in the same study in healthy children, and further, included a separate child and parental proxy-report of HRQoL. In the current cross-sectional design-based study, HRQoL was compared against lifestyle factors in elementary school-aged children from the ages of 6 to 13 years. The hypothesis was that health-promoting lifestyle habits would be related to better HRQoL in the reports of children and their parents. In aging societies the health of children is an even more important factor for future and needs to be followed up on regularly.

The aims of this study are to measure elementary school-aged children’s HRQoL, to compare children’s and the parental reports of their child‘s HRQoL, and to model the lifestyle factors including diet quality, physical activity, screen time, sleep duration, as well as the child’s and the parents’ body mass index in association with the child’s and parents’ proxy report of HRQoL.

## 2. Materials and Methods

### 2.1. Subjects

In this cross-sectional study, a random sample of 5000 families was approached through the Finnish Population Information System, as well as through their hobbies and schools. Information about the study was mailed and posted to the parents, with the invitation to participate in the study sent between March 2017 and February 2018. Parents contacted the investigators via telephone or email if they were interested to participate in this study, and the study visit was arranged. The inclusion criteria were that children were elementary school aged, from grades 1 to 6, from eastern Finland (Kuopio) and southwest Finland (Turku) areas. In Finland, basic education is provided for all youngsters between 7 and 15 years (grades 1 to 6 in elementary school and grades 7 to 9 in upper elementary school). Pre-primary education starts one year before basic education at the age of 6. The children and their families had to have sufficient skills in the Finnish language in order to fill in the questionnaires. Exclusion criteria were: inability to give informed consent for the study, not able to fill in the questionnaires, and parents’ young age (age <18 years). Children with a chronic or serious disease (cancer, surgical treatment) or disability, multiple food allergies or those adhering to a special diet (gluten-free diet, vegan diet) were also reasons of exclusion (except one child with stable type 1 diabetes was included). The study design and participants have been previously described in detail [19].

### 2.2. Measurement of HRQoL

The measurements of HRQoL were made with the Pediatric Quality of Life Inventory^TM^ 4.0 (PedsQL^TM^ 4.0) Generic Core [20], which is a validated measure in this age group of children in Finland [6,21,22]. The PedsQL^TM^ 4.0 comprise parallel child self-report and parental proxy-report formats. The child’s self-reports were inquired of children aged 5–7 and 8–12 [20,23]. The parental proxy report was targeted at parents with children aged 5–7 and 8–12 years and assessed the parents’ perceptions of their child’s HRQoL. The items in the children’s forms are identical, differing only in the questions being framed in developmentally appropriate language. The instructions inquire how much of a problem each item has been during the past month. In the child’s self-report for ages 8–12 and parental proxy reports, a five-point response scale is utilised (never, almost never, sometimes, often, almost always). For the younger children (ages 5–7), the response scale is a three-point scale (not at all, sometimes, a lot of), with each response choice anchored to a happy to a sad face scale [20,23]. PedsQL^TM^ 4.0 consists of 23 items which assess physical functioning (8 items), emotional functioning (5 items), social functioning (5 items) and school functioning (5 items). Items were reverse-scored and converted into a 0–100 scale. Scores were calculated as the sum of the items and divided by the number of items answered (scores were not calculated if more than 50% of the items were missing). There is one total scale score that describes the total HRQoL, four subscales for physical, emotional, social and school functioning, and two summary scores—physical and psychosocial health. Higher scores indicate better HRQoL [20].

### 2.3. Diet Quality

Diet quality was measured with the elementary school-aged Children’s Index of Diet Quality (ES-CIDQ). This index has been developed in the context of this study and was based on the food consumption evaluated by a Food Frequency Questionnaire (FFQ) and nutrient intakes calculated from a 5-day food diary. The development process of this index has been described in detail [19]. The total ES-CIDQ score ranges from 0 to 16.5 points. The overall diet quality was defined as poor (score <6 points) or good (score ≥6 points). The criteria for considering a diet to be health-promoting included the intake of sucrose, saturated fatty acid intake, dietary fiber, vitamin C, calcium, zinc and amounts of vegetables, fruits, and berries consumed in accordance with the national diet recommendations [19].

### 2.4. Physical Activity

During the visit, the leisure-time physical activity (LTPA) was assessed by a self-administered questionnaire. Children and their parents were asked about the frequency of participation in physical activity outside school or working hours and its average intensity and duration of habitual LTPA [24]. The questionnaire has been described in detail [25]. LTPA was calculated as a multiple of the resting metabolic rate (metabolic equivalent [MET] h/week) by multiplying the frequency, mean duration, and mean intensity of weekly LTPA [26]. LTPA was categorised into 3 different activity levels: low (<5 MET h/week, i.e., 10 min/d of moderate-intensity physical activity), moderate (≥5 to ≤30 MET h/week, i.e., physical activity more than 1 h weekly but less than 1 h daily) and active (>30 MET h/week, i.e., 1 h of moderate-intensity physical activity daily) [27].

### 2.5. Screen Time

Time spent looking at a screen was inquired with a question: On average, how many hours per day did the child spend viewing a laptop, computer, television, tablet or play console in a day in the preceding week including weekend? The answers were categorised into three groups in a data-driven manner (≤2 h/d, 2.5 to 4 h/d, >4 h/d).

### 2.6. Sleep Duration

The length of night-time sleep was inquired by the question: How many hours per night did the child sleep on weekdays and weekends during the previous week? Three separate categories were formed based on the mean length of sleep (<9 h/day, 9–10 h/day, >10 h/day).

### 2.7. Anthropometric Measurements and Background Information

Height and weight were measured as described earlier [19]. The body mass index standard deviation score (BMI SDS) was calculated according to the Finnish growth reference curves [28]. Children were categorised as normal weight (including underweight), overweight, or obese. Family background information was inquired with a questionnaire. This included mother’s and father’s ages, weights, and heights, to allow their BMI values to be calculated.

### 2.8. Ethics

The study was conducted according to the guidelines of the Declaration of Helsinki, and the protocol was approved by the Ethics committee for Human Sciences of the University of Turku in 2017 (statement 3/2017). All parents and children provided written informed consent prior to participation.

### 2.9. Statistical Analyses

Background information and HRQoL scores were reported as medians (Q1, Q3) because of the skewness of the distribution. Chi-square or Fisher’s exact test for categorical variables were used to test if there were any differences between the background information of the different age groups. Spearman rank correlations were used to examine the correlations between children’s and parents’ reported HRQoL scores and the measured continuous variables. Mann–Whitney U test was used to evaluate the differences between genders and the related-samples Wilcoxon signed-rank test applied when comparing between the children’s and parents’ evaluation of the HRQoL scores.

The association between HRQoL scores and relevant background factors (town, gender, age as categorised, BMI, ES-CIQO, physical activity (MET, categorised), screen time (categorised) and sleep duration (categorised) were first examined using one-way analysis of variance/covariance. From these analyses, significant explanatory variables were taken into starting multivariable models (linear model), as well as interactions with the age category. The multivariable model was then simplified step-by-step by removing non-significant terms from the statistical model. If the effect included more than two categories, a pairwise comparison was created between categories and these pairwise comparisons were adjusted with Tukey’s method. Since the observed distributions of all the scores showed clear deviations from a normal distribution (most of children/adults had a high quality of life), a special transformation was required to satisfy the assumptions of the linear models. First, we created a ‘mirror’ distribution by subtraction of (101—score) in order to obtain a right-skewed distribution from which the square root was calculated to achieve a normal distribution. In addition, the Spearman correlation was calculated between the scores. In all analyses, a significance level of 0.05 (two-tailed) was used. The data analysis for this paper was generated using SAS software, Version 9.4 of the SAS System for Windows (SAS Institute Inc., Cary, NC, USA) and IBM SPSS Statistics version 28 for Windows (IBM Corp, Armonk, NY, USA).

## 3. Results

### 3.1. Background Information of the Children and Their Parents

The data were obtained from 270 primary-school-aged children from each school class (1st 20.7%, 2nd 21.1%, 3rd 20%, 4th 13.3%, 5th 13.3% and 6th 11.5%) with a median age of 9.7 years (range 6.8–13.4), who were 47% female (Table 1). Most of the children were normal weight. The younger group (6–7 years) included 18.9% of the children with the remaining 81.1% of the children in older group (8–13 years). Diet quality measured with ES-CIDO was a median of 5.9 points (Q1: 4.0, Q3: 8.0); LTPA a median of 31.3 MET (19.5, 35.0); screen time a median of 1 h/day and sleep duration 9.5 h/night. Data were grouped based on the child’s age and the PedsQL-age specific measure (Table 1). Differences between younger and older children were detected in age, diet quality, LTPA, screen time, sleep duration, mother’s age, and father’s age.

### 3.2. Children’s Reports of HRQoL

Younger children reported lower HRQoL than the older children (Table 2). This was detected in all the subscales of PedsQL (all *p* = 0.001). The lowest scores in the younger children were evident in the psychosocial health and its subscales. Older children reported the lowest scores with respect to their emotional functioning. Furthermore, the scores differed between the sexes, i.e., the total score and the subscales for physical and psychosocial health were higher in girls.

### 3.3. Comparison of Child and Parent Proxy-Reports of HRQoL

Mothers were responsible for giving the parent proxy-report of their child’s HRQoL in 93.8% of the cases, and the mother and father together in less than 7% of the cases. Generally, the parents rated their child’s HRQoL better than the younger children themselves but similar as the older children (Table 2). The differences between younger children’s and parent proxy-reports were significant in the total score, and the subscales of physical health, psychosocial health, and school functioning. Here, negative means that the parent reported the subscale as better than the child. Emotional functioning was the only subscale rated better by the older children than by their parents. A better agreement between the child and their parents was detected in the group of older children. Correlations between the child’s and parent proxy-reports were modest in the younger children (Table 3). The strongest correlation was detected in the parental reported total score and the subscales of their child’s physical health, the assessments by both the parent and the child about their physical health and the evaluation by the parent and child on the school functioning subscale. The correlations in the older children’s were also rather weak but nonetheless statistically significant and were related to all subscales of PedsQL with a range of 0.19 to 0.49 (Table 4). The strongest associations were detected between the parent’s and the child’s total score, and the following subscales: parent’s total score and the child’s psychosocial health; parent’s total score and the child’s school functioning; parent’s physical health and the child’s total score; parent’s and the child’s physical health; parent’s psychosocial health and the child’s total score; parent’s and the child’s psychosocial health; and finally, parent’s and the child’s school functioning score.

### 3.4. Children’s Reports of HRQoL and Associated Lifestyle Measures

In the univariate models (Table 5), a better PedsQL total score was significantly associated with female sex, older age, a higher categorised BMI, high physical activity in comparison with low or moderate physical activity and less screen time. The values of ES-CIDQ and sleeping durations were not statistically significantly related to the total score or any of the subscales. Physical health score was significantly associated with older age, a higher BMI, and high physical activity in comparison with children with low or moderate physical activity.

In the multivariable models (Table 5), a higher PedsQL total score was linked to the female gender, older age, high physical activity in comparison with low or moderate physical activity and less screen time (≤2 h/d). The impact of physical activity in this sample was not linear.

The higher physical health score was explained by older age and high physical activity in comparison with low or moderate physical activity. Furthermore, the psychosocial health score was explained by female gender, older age, high physical activity, and less screen time (≤2 h/d).

### 3.5. Parental Reports of HRQoL and Associated Lifestyle Measures

In the univariate models mother´s higher BMI was linked to better parent -proxy- report of total PedsQl total score, physical score, and psychosocial health (Table 6). In the multivariable models better PedsQL total score was explained by a child´s female gender, older age, high physical activity, and less screen time (≤2 h/day). Physical health was associated with the female gender, older age, moderate or high physical activity, and less screen time (≤2 h/d). Psychosocial health was explained by female gender, older age, and high physical activity (Table 7). In children’s reports psychosocial health score was associated with female sex, older age, higher categorised BMI and less screen time (Table 7).

## 4. Discussion

The results revealed that a significantly better HRQoL was reported by 8–13-year-old children (older) in comparison with the 6–7-year-old children (younger). Parents assessed their child’s HRQoL better than the younger children themselves in all the subscales of PedsQL^TM^ 4.0, but about the same as the older children. Female gender, the child’s older age, high physical activity level, and a low amount of screen hours (≤2 h/day) were significant predictors of a better HRQoL in the children’s report and high physical activity, and less screen time (≤2 h/day) in the parental proxy-reports. At odds with our working hypothesis, sleeping and diet were not associated with HRQoL.

The HRQoL total score was a median of 73.9 (mean 73 (SD 11.0)) in the younger children (6–7 years) and a median of 84.8 (mean 83.1 (SD 11.3)) in their older counterparts (8–13 years). In the Finnish validation study of PedsQL^TM^ 4.0 conducted in 8–12-year-old children in the fourth-grade pupils, the mean of the total score was 81.54 (11.46) [21] and this remained almost the same, i.e., a mean of 80.96 (11.76) at the age of ten years [22]. Nonetheless, the follow-up revealed that HRQoL values increased to a mean of 85.1. (10.1) as children grew from age 10 to 12 years. These values are close to those measured in this study. The proposed average HRQoL score measured in healthy populations all around the world is a mean of 80.9 (SD 12.6); the European reference mean score was 80.3 (SD 8.3) with a range of 70.6 to 86.1 [10]. It has been shown that children below the age of seven and above the age of 12 have higher scores [10]. This contrasts with our study where older children had better HRQoL. Similar results were measured in a study which compared children of three ages, ages 6–8, 9–11 and 12–17, of which the oldest age group exhibited a decline in the HRQoL domains [16]. A decrease in HRQoL has been found as Chinese children grow older from 9 to 17 years of age [29]. The differences in the reports between younger and older children may reflect the fact that elementary school-aged children in the first and second grades are still not mature enough to be able to reliably report their HRQoL. However, the developer of this instrument has demonstrated that 5-year-old children can reliably and validly self-report their HRQoL with an age-appropriate instrument [5]. However, if younger children are not asked to fill in the questionnaires, this may potentially have affected their reports.

The subscales that were reported with the highest values were physical health both in younger and older children, and social functioning in older children. The results support previous findings both from Finland and elsewhere in which children reported the highest subscales of physical health [6,16,22] and social functioning [6,16,22]. The lowest scores in younger children were encountered with psychosocial health and its subscales. Older children reported their emotional functioning as the worst, as has been the case in the earlier studies [6,16,20].

A better HRQoL was seen in girls in comparison with the boys when we assessed total, physical, psychosocial, emotional, and school functioning, but no differences were found in social functioning. In one report, Finnish 10-year-old girls reported lower emotional functioning than the boys [22]. In comparison, at the age of 10 to 12 years, all HRQoL scores improved, with the boys reporting significantly better HRQoL [6,30]. There are differences between the Finnish studies conducted over 10 years ago by Laaksonen and our study, i.e., the children that they examined were 4th grade boys and girls, whereas in our study, children were elementary school-aged; nonetheless, the results are rather similar.

We compared and demonstrated that children’s and parental correlations of the HRQoL subscales were higher in the group of older children and this finding is supported by earlier publications [6,7]. Differences between children’s and their parents’ reports of HRQoL were statistically significant in the total, physical, and psychosocial health, and school functioning in the younger children, and between emotional and school functioning in the older children. Younger children´s parents reported higher HRQOL than the children themselves. Varni [23] detected a trend towards higher inter-correlations between the parent’s and child’s reports as the child became more mature, which was seen in our study. In the Finnish study, 10-year-old children reported higher HRQoL scores than parents in the following domains—physical, emotional, school and social—but lower in social and school functioning than the assessment of the parents [6]. HRQol increased in both the children’s and parents’ reports with age [6]. The child’s age and domains of the measure and the parents’ own HRQoL may affect the agreement between how the parent and their child assesses the HRQoL [7].

From all of the measured lifestyles, only high physical activity and less screen time were significantly related to HRQoL in both children’s and parent’s reports, and child’s age and gender in the children’s reports. Parallel results have been reported where HRQoL physical health and psychosocial health were positively associated with a sufficient sleep duration and moderate/vigorous activity and negatively correlated with screen time [16]. A systematic review found evidence that higher levels of physical activity were associated with higher HRQoL, whereas more sedentary behaviours were linked with lower HRQoL [17]. Sedentary behaviour was characterised as screen-based activities such as watching television, using smartphones, and playing computer games. Physical activity and screen time were self-reported in most of the studies and HRQoL was measured with seven different HRQoL instruments in children aged 3 to 18 years [17]. Very few studies on healthy children have included how the child assessed their HRQoL in association with lifestyle measures including diet, physical activity, screen time, sleeping duration and BMI [29,31]. Children from 12 countries were compared and HRQoL was assessed with the KIDSCREEN-10 measure of HRQoL. A cluster of healthy lifestyle behaviours in 9–11-year-old children was found, i.e., low screen time, a healthy eating pattern based on a food frequency questionnaire, moderate physical activity, and moderate sedentary behaviour [31]. Most often either physical activity and screen time or physical activity and diet quality have been measured [30,32].

Leisure-time physical activity was low, 6.9%, moderate, 35.4%, and high, 57.7% in the children. In the models, differences were detected in comparisons between low activity versus high activity, and moderate versus high activity groups. It is difficult to compare across studies because of the different physical activity measures being applied. Some measurements have been performed with self-reported questionnaires, and others with accelerometers which give a very accurate picture of daily physical activity. Nonetheless, we believe that the children in our study were physically active. Wong [16] used the International Physical Activity Questionnaire (IPAQ) and grouped the physical activity of 6–17-year-old children in the following manner: low, 2.6%, moderate, 29.9%, and the majority, i.e., 67.4%, highly active.

We assessed screen time as viewing a laptop, computer, television, tablet or play console and the median time spent was one hour per day. A total of 67.4% of the children spent ≤2 h/day on these activities, a value that is much less than in some reports for gaming and leisure activities where durations of 3.1 h per day have been described in children aged 6 rising up to 3.7 h in 11 year-olds [16]. Another study reported values of under 2 h per day in 96.8% and over 2 h per day in 3.2% of primary school-aged children [29]. Better HRQoL was associated with less than 1–2 h per day spent on screen-based activities in that study.

Usually, diet is not included in lifestyle measures. The ES-CIDQ-index was used to measure the quality of a diet but no association with HRQoL was found, although there was a difference between younger and older children, i.e., younger children had healthier food choices. One explanation may be the fact that the PedsQL^TM^ 4.0 does not include questions that are connected to diet or eating behaviour; it is really intended to assess physical, emotional, social functioning and school functioning, and thus wellbeing in general. It is possible that an association may become evident in large samples. In a systematic review and meta-analysis of published studies including healthy children and adolescents, a high-diet quality, and healthy dietary behaviours were associated with increased HRQoL [18]. The studies included in that review had applied various measures of HRQoL. In a recent systematic review, adherence to a Mediterranean diet was explored. The researchers concluded that there was a positive correlation between how well the children and adolescents consumed a Mediterranean diet and their HRQoL values. However, only four out of eleven studies were assessed as having a low risk of bias, Mediterranean diet adherence was assessed with the KIDMED index or Krece Plus test, and the ages of children have varied from 6 to 18 years. Most studies have been conducted in South European countries [14,33]. In a recent study, children were grouped on the basis of HRQOL as measured with the EuroQol-5 Dimensions-5 levels (EQ-5D-5L) validated questionnaire and adherence to a Mediterranean diet was strongest in the 70.8% of those children with the highest HRQoL [34]. In a Canadian study, children with healthier eating patterns had higher HRQoL, and unhealthy eating patterns were linked to lower HRQoL [15].

We hypothesised that there would be an association between HRQoL and diet because earlier studies have shown HRQoL to be worse in overweight and obese children. The child’s categorised BMI (i.e., as normal weight, overweight, obese) was an important predictor in the child´s reports with respect to the total PedsQL score, physical health, and psychosocial health in univariate models. A similar phenomenon was detected in mothers’ BMI as a continuous variable in relation to parental proxy-report of the PedsQL score, physical health, and psychosocial health. The sample included 15.9% overweight and 6.2% obese children. The HRQoL was lower in the obese children than in normal weight children. Nonetheless, in the multivariate models, this relationship disappeared. Research has shown that overweight and obese children with BMI values above normal have significantly lower total, physical, and psychosocial HRQoL [11,13] and their parents reported even more greater HRQoL reductions than the children themselves [11]. This association has not been observed in all studies [34].

There are many strengths in this study. Firstly, the objective was to examine children from each of the school grades (1 to 6) evenly; unfortunately, this was not fully realised as the number of children was lower in the older grades. One explanation may be that the age of 11 to 12 is the time of prepuberty, when children’s willingness to cooperate may be reduced. Secondly, the HRQoL reports were obtained from both parents and children. Thirdly, an extensive battery of lifestyle measures was included: diet, physical exercise, sleep duration, screen time, with the measurements made with validated instruments. Fourthly, we had national reference data for comparison from an earlier national HRQoL validation study in elementary school-aged children which unfortunately did not assess lifestyle measurements.

The limitations of this study were, firstly, that it was cross-sectional. Secondly, some selection bias may have occurred favouring families with healthier lifestyles when families were approached through the Finnish Population Information System, as well as through hobbies and schools, and the sample was quite small. Community samples have been shown to have better HRQoL values. The ability of the children in the early school years to complete HRQoL life instruments may be somewhat limited. However, the PedsQL instrument has been validated and widely used in studies with the ages of the respondents ranging from infants aged 5- to 18-year-old adolescents. Thirdly, the lifestyle measures were self-reported and screen time, sleeping duration, and LTPA were collected by self-reported questionnaires. Seasonal variation and puberty were not taken into account in the data collection.

Though there has been increasing interest in measuring HRQoL in recent years, systematic review and meta-analyses are needed to collect and evaluate the data. It has been challenging to draw definitive conclusions because of the different ways that HRQoL can be measured, some of which are not entirely comparable [35], and there have also been different measures of physical activity, diet, and screen time. Cultural differences exist and these introduce challenges when comparing studies from different countries. Repeated studies using the same measures are needed to confirm the results and measure the change with time in various cultural contexts and children in different age-groups separately. The comparability between studies would increase if one were to conduct two different measures of HRQoL in the same study. More longitudinal and repeated studies are needed to confirm the age- and gender-dependent differences observed here.

Means to promote healthy lifestyles should be particularly targeted to young children and boys. It would be important to find child-friendly ways to promote physical activity, as well as free-time activities which would compete with the enticements of the computer screen and the smart phone. In fact, these may represent means to reduce sedentary behaviours at the societal level. In Finnish studies, the HRQoL has been high, and subscales have been rated in the best categories despite the changes that have happened in broader society. HRQoL and subscale-related differences between sexes and lifestyle measures in healthy children reveal areas for health promotion. New and varied approaches are needed in societies to prevent the rising trend of overweight and obesity, and the development of inequalities. As populations are aging and the birth rates are declining, this is more important than ever before.

## Figures and Tables

**Table 1:** Characteristics of children and their parents.

	All Children n (%)	Age 6–7 Years n (%)	Age 8–13 Years n (%)	*p-*Value
Children’s characteristics	270	51 (18.9)	219 (81.1)	
Gender, female	127 (47)	23 (45.1)	104 (47.5)	0.76 ^a^
Age, years, median (Q1, Q3)	9.7 (8.4, 11.2)	7.4 (7.1, 7.8)	10.0 (8.9, 11.5)	
BMI SDS	270	51	219	0.22 ^b^
Normal weight	213 (78.9)	37 (72.5)	176 (80.4)	
Overweight	43 (15.9)	9 (17.6)	34 (15.5)	
Obese	14 (5.2)	5 (9.8)	9 (4.1)	
Diet quality (ES-CIDQ)	263	48	215	0.029 ^c^ 0.052 ^a^
Median (Q1, Q3)	5.9 (4.0, 8.0)	6.5 (5.0, 8.5)	5.5 (4.0, 8.0)	
Good diet quality	131 (50.2)	30 (62.5)	101 (47.0)	
Poor diet quality	132 (49.8)	18 (37.5)	114 (53.0)	
Leisure-time physical activity	260	46	214	0.14 ^c^ 0.083 ^b^
MET, median (Q1, Q3)	31.3 (19.5, 35.0)	19.5 (19.5, 35.0)	31.3 (19.5, 52.2)	
Low activity	18 (6.9)	2 (4.3)	16 (7.5)	
Moderate activity	92 (35.4)	23 (50.0)	69 (32.2)	
High activity	150 (57.7)	21 (45.7)	129 (60.3)	
Screen time, h/day	261	46	215	0.001 ^c^ 0.001 ^b^
Median (Q1, Q3)	1 (1.0, 2.0)	1 (1.0, 1.0)	1.5 (1.0, 2.0)	
≤2 h/day	176 (67.4)	44 (93.6)	132 (61.7)	
2.5–4 h/day	63 (24.1)	1 (2.1)	62 (29.0)	
>4 h/day	22 (8.4)	2 (4.3)	20 (9.3)	
Sleep duration	261	47	214	0.001 ^c^ 0.001 ^b^
Median (Q1, Q3)	9.5 (9.0, 10.0)	10 (10.0, 10.5)	9.8 (9.0, 10.0)	
<9 h	66 (25.3)	4 (8.5)	62 (29.0)	
9–10 h	159 (60.9)	28 (58.7)	131 (61.2)	
>10 h	36 (13.8)	15 (31.9)	21 (9.8)	
Living area	270	51	219	0.32 ^a^
Eastern Finland	90 (33.3)	20 (39.2)	70 (32)	
Southwest Finland	180 (66.7)	31 (60.8)	149 (68)	
Parents’ characteristics				
Age, mother	261	47	214	0.001 ^c^
Median (Q1, Q3)	41.0 (37.0, 44,0)	38.0 (35.0, 43.0)	41.5 (37.0, 44.0)	
Age, father	253	47	206	0.005 ^c^
Median (Q1, Q3)	42.0 (39.0, 47,0)	40.0 (37.0, 44.0)	43.0 (39.8, 47.0)	
BMI, mother	253	45	208	0.66 ^c^
Median (Q1, Q3)	23.6 (21.9, 27.2)	24.1 (21.9, 27.9)	23.6 (21.9, 26.9)	
BMI, father	233	44	189	0.79 ^c^
Median (Q1, Q3)	25.1 (23.8, 28.1)	25.6 (23.8, 28.4)	25.3 (23.9, 28.1)	

^a^ Pearson chi-square test. ^b^ Fisher exact test. ^c^ Independent samples Mann–Whitney U test.

**Table 2:** Children’s and parental proxy-report of HRQoL.

PedsQL Score	Child Self-Report		Parent Proxy-Report		Difference (Child–Parent)		*p-*Value ^a^
	Md (Q1, Q3)	M (SD)	Md (Q1, Q3)	M (SD)	Md (Q1, Q3)	M (SD)	
Children 6–7 years (n = 51)							
Total Score	73.9 (65.2, 82.6)	73.0 (11.0)	78.3 (72.8, 82.6)	77.2 (9.6)	−4.3 (−14.1, 4.3)	−4.2 (11.5)	0.017
Physical Health	81.3 (68.8, 87.5)	76.2 (12.7)	81.3 (71.9, 87.5)	79.9 (12.2)	−3.1 (−12.5, 3.1)	−3.7 (13.0)	0.036
Psychosocial Health	70.0 (63.3, 80.0)	71.2 (11.5)	76.7 (68.3, 82.8)	75.8 (10.0)	−4.2 (−15.0, 5.0)	−4.5 (12.7)	0.020
Emotional Functioning	70.0 (60.0, 80.0)	67.8 (15.4)	75.0 (65.0, 80.0)	72.4 (11.0)	−5.0 (−15.0, 10.0)	−4.5 (17.4)	0.091
Social Functioning	70.0 (62.5, 90.0)	75.1 (14.2)	75.0 (65.0, 85.0)	76.0 (14.4)	0.0 (−15.0, 10.0)	−0.9 (17.8)	0.78
School Functioning	70.0 (60.0, 80.0)	71.1 (16.3)	80.0 (70.0, 90.0)	79.0 (13.4)	−10.0 (−15.0, 1.3)	−7.9 (16.2)	0.003
Children 8–13 years (n = 219)							
Total Score	84.8 (76.1, 92.4)	83.1 (11.3)	84.8 (76.1, 90.2)	83.4 (9.0)	0.0 (−6.5, 5.8)	−0.3 (10.5)	0.95
Physical Health	87.5 (78.1, 93.8)	84.1 (11.7)	87.5 (81.3, 93.8)	85.3 (10.4)	0.0 (−6.3, 6.3)	−1.2 (11.1)	0.22
Psychosocial Health	85.0 (75.0, 91.7)	82.5 (12.6)	83.3 (75.0, 90.0)	82.3 (10.2)	0.0 (−8.3, 7.9)	0.2 (12.2)	0.63
Emotional Functioning	80.0 (65.0, 90.0)	76.2 (15.9)	75.0 (60.0, 85.0)	73.4 (14.2)	5.0 (−10.0, 15.0)	2.9 (16.5)	0.009
Social Functioning	95.0 (80.0, 100.0)	89.2 (12.9)	95.0 (85.0, 100.0)	89.5 (11.7)	0.0 (−5.0, 10.0)	−0.3 (14.3)	0.95
School Functioning	85.0 (70.0, 95.0)	82.0 (14.6)	85.0 (75.0, 95.0)	84.0 (11.9)	0.0 (−10.0, 5.0)	−2.0 (14.2)	0.041

^a^ Related samples Wilcoxon signed-rank test.

**Table 3:** Spearman correlations between the child’s (6–7 years) (n = 51) and the parent proxy-report of HRQoL.

	Child					
Parent	Total Score	Physical Health	Psychosocial Health	Emotional Functioning	Social Functioning	School Functioning
Total Score	0.35 *	0.42 **	0.27	0.26	0.09	0.26
Physical Health	0.34 *	0.46 **	0.21	0.18	0.11	0.10
Psychosocial Health	0.29 *	0.33 *	0.25	0.21	0.08	0.29 *
Emotional Functioning	0.03	0.14	−0.03	0.12	−0.22	0.01
Social Functioning	0.26	0.29 *	0.24	0.13	0.21	0.16
School Functioning	0.32 *	0.30 *	0.29 *	0.21	0.07	0.43 **

* Correlation is significant at the 0.05 level, ** Correlation is significant at the 0.01 level.

**Table 4:** Spearman correlations between the child’s (8–13 years) (n = 219) and the parent proxy-report of HRQoL.

	Child					
Parent	Total Score	Physical Health	Psychosocial Health	Emotional Functioning	Social Functioning	School Functioning
Total Score	0.49 **	0.38 **	0.48 **	0.38 **	0.39 **	0.45 **
Physical Health	0.45 **	0.48 **	0.39 **	0.33 **	0.32 **	0.35 **
Psychosocial Health	0.43 **	0.27 **	0.45 **	0.35 **	0.36 **	0.42 **
Emotional Functioning	0.39 **	0.25 **	0.41 **	0.39 **	0.32 **	0.28 **
Social Functioning	0.33 **	0.16 *	0.36 **	0.27 **	0.32 **	0.31 **
School Functioning	0.35 **	0.25 **	0.35 **	0.19 **	0.25 **	0.47 **

* Correlation is significant at the 0.05 level, ** Correlation is significant at the 0.01 level.

**Table 5:** Associations between the child’s reported PedsQL scores and lifestyle factors. A linear model was first performed for each of the lifestyle factors separately and then a multivariable model was built for all significant factors. Subsequently, a multivariable model was simplified by removing non-significant terms one-by-one. The final model is presented in the table below. When a factor was statistically significant, then a pairwise testing between all categories was performed and *p*-Values were corrected with Tukey’s method. Transformation was necessary for both the total score and subscales in order to achieve the assumption of normality. First, subtraction from 101 was calculated (where 100 was the maximum score for each of the scores) and then a square root transformation was applied.

		Univariate Model ^a^		Multivariable Model	
	Lifestyle Factors	*p*-Value	Adj. *p*-Value	*p*-Value	Adj. *p-*Value
Total score	Gender	0.0004		<0.0001	
	Age	<0.0001		<0.0001	
	**BMI, categorised**	0.008			
	normal weight vs. overweight		0.37		
	normal weight vs. obesity		0.030		
	overweight vs. obese		0.023		
	**Physical activity**	0.0003		0.001	
	low vs. moderate		0.25		0.19
	low vs. high		0.003		0.0044
	moderate vs. high		0.006		0.029
	**Screen time, h/day**	0.026		0.0006	
	≤2 h vs. 2.5–4 h		0.11		0.0042
	≤2 h vs. >4 h		0.086		0.014
	2.5–4 h vs. >4 h		0.74		0.80
Physical health	Gender	0.067			
	Age	<0.0001		0.0002	
	**BMI, categorised**	0.038			
	normal weight vs. overweight		0.10		
	normal weight vs. obesity		0.03		
	overweight vs. obese		0.40	0.0005	
	**Physical activity**	0.0003			
	low vs. moderate		0.20		0.094
	low vs. high		0.0027		0.0016
	moderate vs. high		0.0094		0.035
	**Screen time h/day**				
	≤2 h vs. 2.5–4 h				
	≤2 h vs. >4 h				
	2.5–4 h vs. >4 h				
Psychosocial health	Gender	<0.0001		<0.0001	
	Age	<0.0001		<0.0001	
	**BMI, categorised**	0.0048			
	normal weight vs. overweight		0.56		
	normal weight vs. obesity		0.042		
	overweight vs. obese		0.0085		
	**Physical activity**	0.0012		0.0038	
	low vs. moderate		0.41		0.32
	low vs. high		0.013		0.016
	moderate vs. high	0.037	0.011		0.045
	**Screen time h/day**			0.0008	
	≤2 h vs. 2.5–4 h		0.15		0.0059
	≤2 h vs. >4 h		0.094		0.015
	2.5–4 h vs. >4 h		0.70		0.77

^a^ Town, sleeping duration, and ES-CIDQ were also tested but they were all nonsignificant in the univariate models.

**Table 6:** Associations between the parental report of PedsQL scores and lifestyle factors.

		**Univariate Model ^a^**		**Multivariable Model**	
	Lifestyle Factors	*p*-Value	Adj. *p*-Value	*p*-Value	Adj. *p-*Value
Total score	Gender			0.0012	
	Age			<0.0001	
	**BMI, categorised**				
	normal weight vs. overweight				
	normal weight vs. obesity				
	overweight vs. obese				
	**Physical activity**			0.0013	
	low vs. moderate				0.96
	low vs. high				0.24
	moderate vs. high				0.0013
	**Screen time**			0.0043	
	≤2 h vs. 2.5–4 h				0.0029
	≤2 h vs. >4 h				0.64
	2.5–4 h vs. >4 h				0.45
	Mother, BMI	0.015			
Physical health	Gender			0.0098	
	Age			0.0077	
	**BMI, categorised**				
	normal weight vs. overweight				
	normal weight vs. obesity				
	overweight vs. obese				
	**Physical activity**			0.0017	
	low vs. moderate				0.46
	low vs. high				0.017
	moderate vs. high				0.014
	**Screen time h/day**			0.017	
	≤2 h vs. 2.5–4 h				0.015
	≤2 h vs. >4 h				0.99
	2.5–4 h vs. >4 h				0.17
	Mother, BMI	0.022			

**^a^** Town, sleeping duration, and ES-CIDQ were also tested but they were all nonsignificant in the univariate models.

**Table 7:** Median values (Q1, Q3) for PedsQL total score, physical health, psychosocial health for significant lifestyle measures.

	Gender		Physical Activity (MET)			Screen Time h/d		
	Female (n = 127)	Male (n = 143)	Low (n = 18)	Moderate (n = 92)	High (n = 150)	≤2 h (n = 176)	2.5–4 h (n = 63)	>4 h (n = 22)
Child’s Report (Md (Q1, Q3)								
Total Score	85.9 (78.3, 92.4)	80.4 (70.0, 87.0)	76.6 (64.4, 84.0)	80.4 (71.9, 87.9)	85.9 (78.3, 92.4)	84.3 (76.1, 92.4)	81.5 (72.8, 89.1)	81.5 (70.7, 84.8)
Physical Health	87.5 (78.1, 93.8)	81.3 (75.0, 90.6)	79.7 (68.8, 85.2)	81.3 (75.0, 90.6)	87.5 (78.1, 93.8)	87.5 (78.1, 93.8)	84.4 (75.0, 90.6)	81.3 (75.0, 87.5)
Psychosocial Health	86.7 (76.7, 93.3)	80.0 (66.7, 88.3)	73.3 (64.2, 83.8)	78.5 (70.0, 88.3)	86.7 (76.3, 93.3)	83.3 (73.1, 91.7)	80.0 (70.0, 88.3)	80.0 (65.8, 89.5)
Parental Report Md (Q1, Q3)								
Total Score	85.9 (77.2, 90.2)	80.4 (73.9, 88.0)	84.8 (69.0, 87.6)	81.0 (73.9, 85.1)	85.9 (78.3, 91.3)	83.7 (77.2, 90.2)	80.4 (71.7, 88.0)	84.8 (76.1, 88.3)
Physical Health	87.5 (81.3, 93.8)	84.4 (78.1, 93.8)	85.9 (60.9, 89.6)	84.4 (78.1, 90.6)	87.5 (81.3, 93.8)	87.5 (81.3, 93.4)	84.4 (75.0, 90.6)	87.5 (83.6, 93.8)
Psychosocial Health	83.3 (75.0, 91.7)	83.3 (75.0, 90.0)	84.8 (71.3, 87.5)	79.2 (71.7, 85.0)	83.3 (76.7, 91.7)	82.5 (75.0, 91.7)	80.0 (70.0, 86.7)	82.5 (73.3, 88.3)

## Data Availability

The data sets are not available due to their containing information that could compromise participant privacy and consent.
